# Application of intraluminal brachytherapy for malignant obstruction in the porta hepatis: a retrospective control study

**DOI:** 10.3389/fonc.2025.1416565

**Published:** 2025-05-01

**Authors:** Yi Zhang, Yang Yang, Lingling Li, Peimin Li

**Affiliations:** ^1^ Department of Interventional Therapy, Heping Hospital Affiliated to Changzhi Medical College, Changzhi, Shanxi, China; ^2^ Department of Neurology, Luzhou Branch of Changzhi People’s Hospital, Changzhi, Shanxi, China; ^3^ Department of Radiology, Shanghai Pulmonary Hospital of Tongji University, Shanghai, China

**Keywords:** malignant obstructive jaundice, portal vein tumor thrombus, stent placement, iodine-125 seed strands, transarterial chemoembolization (TACE)

## Abstract

**Purpose and background:**

Malignant obstructions in the porta hepatis mainly include malignant obstructive jaundice (MOJ) and portal vein tumor thrombus (PVTT). Stent placement has been one of the most commonly recommended methods to alleviate the physical suffering of these patients, but the long-term outcome has been frustrating in terms of stent occlusion. The aim of this study was to discuss the clinical effect and technical feasibility of intraluminal brachytherapy (ILBT) in patients with malignant obstruction in the porta hepatis

**Methods and materials:**

From 2016 to 2018, 68 patients diagnosed with malignant obstruction in the porta hepatis were retrospectively included in this study. Twenty-eight patients (group A) received stent placement with iodine-125 seed-strand implantation, and 40 patients (group B) received stent placement only. All patients underwent numerous transarterial chemoembolizations (TACE) after stent implantation. All patients were followed up until death. Clinical data, stent patency and survival time were recorded for further analysis.

**Results:**

There was no significant difference between the two groups in terms of length of malignant obstruction and baseline characteristics. 68 stents were successfully implanted in both groups.Iodine-125 seed strands were successfully deployed and completely covered the length of the stent in group A. Liver function and jaundice improved continuously in the first 9 months after treatment (P<0.05). Compared to group B, the mean stent patency time was significantly longer in group A (5.5 ± 2.09 months versus 6.86 ± 1.82 months, P<0.001). The mean survival time is longer in group A than in patients in group B (10.03 ± 3.04 months VS 7 ± 2.44 months, P<0.001).

**Conclusion:**

ILBT in combination with stent implantation and TACE has proven to be a feasible and effective palliative treatment to maintain stent patency in patients with PVTT and MOJ.

## 1 Introduction

Malignant tumors in the porta hepatitis can include hepatocellular carcinoma (HCC), extrahepatic cholangiocarcinoma, pancreatic head carcinoma, and liver metastases. They can cause obstruction of the portal vein or bile ducts, which then leads to malignant obstructive jaundice (MOJ) and portal vein tumor thrombus (PVTT) (1–3). These malignant obstructions could not be easily resolved by surgical resection and may have had a poor prognosis due to the increased risk of tumor spread, variceal hemorrhage, ascites, jaundice, hepatic encephalopathy and liver failure (3–7).

Stent implantation was a safe palliative therapy that was able to achieve patency of the lumen and had significantly fewer serious adverse effects (5, 8). However, occlusion of the inserted stent due to tumor progression and growth can lead to recurrence of jaundice or increased portal pressure (9, 10) However, the placement of a stent does not treat the tumor. Some previous studies have reported that both transarterial chemoembolization (TACE) and brachytherapy are effective treatments for local control of the tumor in the hepatic hilar region (11–17). In the study presented here, we suggested that a combined treatment of stent implantation, implantation of iodine-125 seed strands and TACE could achieve local control of the malignant tumor and prolonged patency of the stent. Some successful experiences have been reported as follows.

## 2 Materials and methods

### 2.1 Patient general information

Between 2016 and 2018, 97 patients with MOJ or PVTT were treated in our department. The evaluation criteria were as follows: (1)the diagnosis of MOJ or PVTT was confirmed by typical imaging findings in combination with tumor markers (e.g.: serum alpha-fetoprotein persistently elevated above 400 ng/ml) or pathology; (2) patients with MOJ or PVTT were unsuitable for resection according to the cancer staging classification; (3) patients had not been previously treated with stent implantation and radiotherapy; (4) the obstruction would require at least a 0.035-inch guidewire; (5) granulocyte count >1.5×109/L, platelet count >85×109/L, serum creatinine level ≤115μmol/L, and prothrombin time was within 3 seconds of control; (6) life expectancy >3 months. 68 patients were eventually enrolled in this study. Of all subjects, 28 patients (group A) received stent implantation, iodine-125 seed-strand implantation and TACE, and the remaining 40 patients (group B) received stent implantation and TACE.

The length of the malignant obstruction was determined on the basis of the results of the CT scans prior to treatment. Laboratory tests such as liver function, kidney function, blood count and coagulation parameters before and 3 days after surgery were recorded.

### 2.2 Surgical procedure

#### 2.2.1 Self-produced iodine-125 seed strands

The diameter and length of the iodine-125 seeds (XinKe, Shanghai, China) were 0.8 mm and 4.5 mm, respectively. The radioactivity of each seed was 25.9 MBq with an irradiation range of 20 mm and a half-life of 59.4 days. The number of iodine-125 seeds was calculated based on the CT scan and the following formula: N = length of obstructive portion of bile duct (mm) or main portal vein (mm)/4.5 + 4 (12, 14). A hyaline catheter with an inner diameter (ID) of4F was used to insert the seeds. One end of the catheter was heated and clamped for sealing. All seeds were arranged linearly and loaded into the sealed catheter, and the redundant part of the other side was cut off and sealed as before. If one catheter was too short to contain all the seeds, multiple catheters could be used for sealing.

#### 2.2.2 Stent placement

MOJ underwent percutaneous transhepatic cholangial drainage (PTCD) one week prior to stent placement to alleviate jaundice. A 0.035-inch hydrophilic guidewire (Terumo Corporation, Tokyo, Japan) was inserted into the distal duodenum through the drainage catheter, and then a 5F sheath (Cook Incorporated, Bloomington, USA) was replaced over the guidewire. Cholangiography was performed to measure the location and length of the obstruction. After measurement, a 0.035-inch stiff wire (Terumo Corporation, Tokyo, Japan) and a 0.018-inch guidewire (Terumo Corporation, Tokyo, Japan) were inserted into the distal duodenum. After removing the 5F protective sheath, the self-expandable nitinol stent of appropriate size (Micro-tech Co.Ltd, Nanjing, China) was inserted over the stiff wire and released to completely cover the edge of the obstructive lesion (both ends of the stent beyond the obstruction ≤10 mm). The 0.018-inch wire was left outside the stent.

For PVTT, after local anesthesia, a 22G Chiba needle (Cook Incorporated, Bloomington, USA) was used to puncture the patent second order branches of the intrahepatic portal vein under ultrasound guidance. Once successful, a 0.018-inch guidewire (Terumo Corporation, Tokyo, Japan) was carefully inserted through the obstruction into the distal splenic vein, and then a 6FNEFF set was inserted over the guidewire. a 4F vertebral catheter (Terumo Corporation, Tokyo, Japan) was inserted to perform angiography in the splenic vein or superior mesenteric vein to reveal the obstruction and measure the pressure. A 5F sheath was exchanged and then a 0.035-inch stiff wire and a 0.018-inch guidewire were inserted into the splenic vein. The appropriate size self-expandable stent was inserted over the rigid wire and completely covered the area of the lesion (both ends of the stent beyond the edge of the lesion ≤ 10mm). 0.018-inch wire was left outside the stent. Keep the rigid wire still and retract the stent delivery system and 5F sheath.

#### 2.2.3 Implantation of iodine-125 seed strands

The outer cannula of the NEFF set was carefully inserted into the distal stent over the 0.018-inch guidewire (outside the stent). The 0.018-inch wire was retracted and the iodine-125 seed strand was loaded into the outer cannula of the NEFF set. The stiffening cannula of the NEFF set was used to bring the strand to the target position, and then slowly and carefully withdrew the outer cannula and the stiffening cannula. If multiple strands were needed, at least two 0.018-inch wires should be inserted and left outside the stent.After implantation, the transhepatic puncture track was closed with an absorbable gelatin sponge (Jinling Pharmaceutical CO.LTD, Nanjing, China).

#### 2.2.4 TACE

After local anesthesia and puncture of the femoral artery, a 5F RH catheter (Terumo Corporation, Tokyo, Japan) was inserted to perform angiography of the celiac boot, superior mesenteric artery and bilateral inferior phrenic artery. Once the feeding arteries were confirmed, the target arteries were super-selectively catheterized using a 2.4 F microcatheter (Terumo Corporation, Tokyo, Japan). Depending on the patient’s liver function and the vascularity of the tumor, 20 ml of a mixture of oxaliplatin, pharmacorubicin and iodinated oil was pumped into the tumor at a rate of 0.5–1 ml/min until stasis flow was achieved in the tumor vessels under fluoroscopic monitoring. The feeding arteries were embolized with an absorbable gelatin sponge. All included patients received a maximum of 4 cycles of TACE in the following 4 months.

### 2.3 Follow up

All patients were followed up until death. During the first 4 months, a CT scan of the abdomen was performed every two months to assess the efficacy of the combined therapy. Laboratory tests, such as liver function, kidney function, blood count and coagulation parameters, were analyzed monthly. On the question of protection from radioactive particles. Patients should wear a 0.25 to 0.5 mm lead-containing abdominal band for 6 months and maintain a distance of at least 1 meter from other people. After 6 months, protection is generally no longer necessary.

### 2.4 Statistical analysis

All results were expressed as mean ± SD and statistical analysis was performed using SPSS21.0 software. Paired-samples t-tests were used to compare the statistical significance of differences. In all analyses, a P value <0.05 was considered statistically significant.

## 3 Results

### 3.1 Baseline characteristics between two groups

Among 28 patients in group A, 13 patients were diagnosed with PVTT and 15 patients with MOJ. In group B, there were 21 patients with PVTT and 19 patients with MOJ (P=0.403). There were no significant differences between the two groups in terms of age, gender, pathological types, duration of obstruction, Chid-Pugh class, ECOG score and previous treatment (**Table 1**).

**Table 1:** Baseline characters in both groups.

Characteristics	Group A	Group B	P value
Type of obstruction (PVTT/MOJ)	13/15	21/19	0.403
Age (years) (M ± SD)	62. 1 ± 11.0	61.4 ± 11.6	0.576
Sex (male/female) Pathologic type	19/9	28/12	0.529
(HCC/cholangiocarcinoma/pancreatic carcinoma/gastric carcinoma/hepatic metastasis)	13/10/3/0/2	18/12/5/2/3	0.805
Length of obstruction (mm)	31.3 ± 7.3	31.5 ± 6.5	0.542
Child-Pugh class (A/B/C)	22/7	23/17	0.092
ECOG Score (0/1/2/3)	9/7/12/0	15/11/13/1	0.716
Previous treatment (None/surgical resection/TACE/combined)	8/3/15/2	11/5/23/1	0.825
TACE procedure	3.4 ± 0.55	3. 11 ± 0.6	0.311

PVTT, portal vein tumour thrombus; MOJ, malignant obstructive jaundice; HCC, hepatocellular carcinoma; TACE, transarterial chemoembolisation; ECOG score, eastern cooperative oncology group score.

### 3.2 Results of surgical procedure

**Figure 1:**
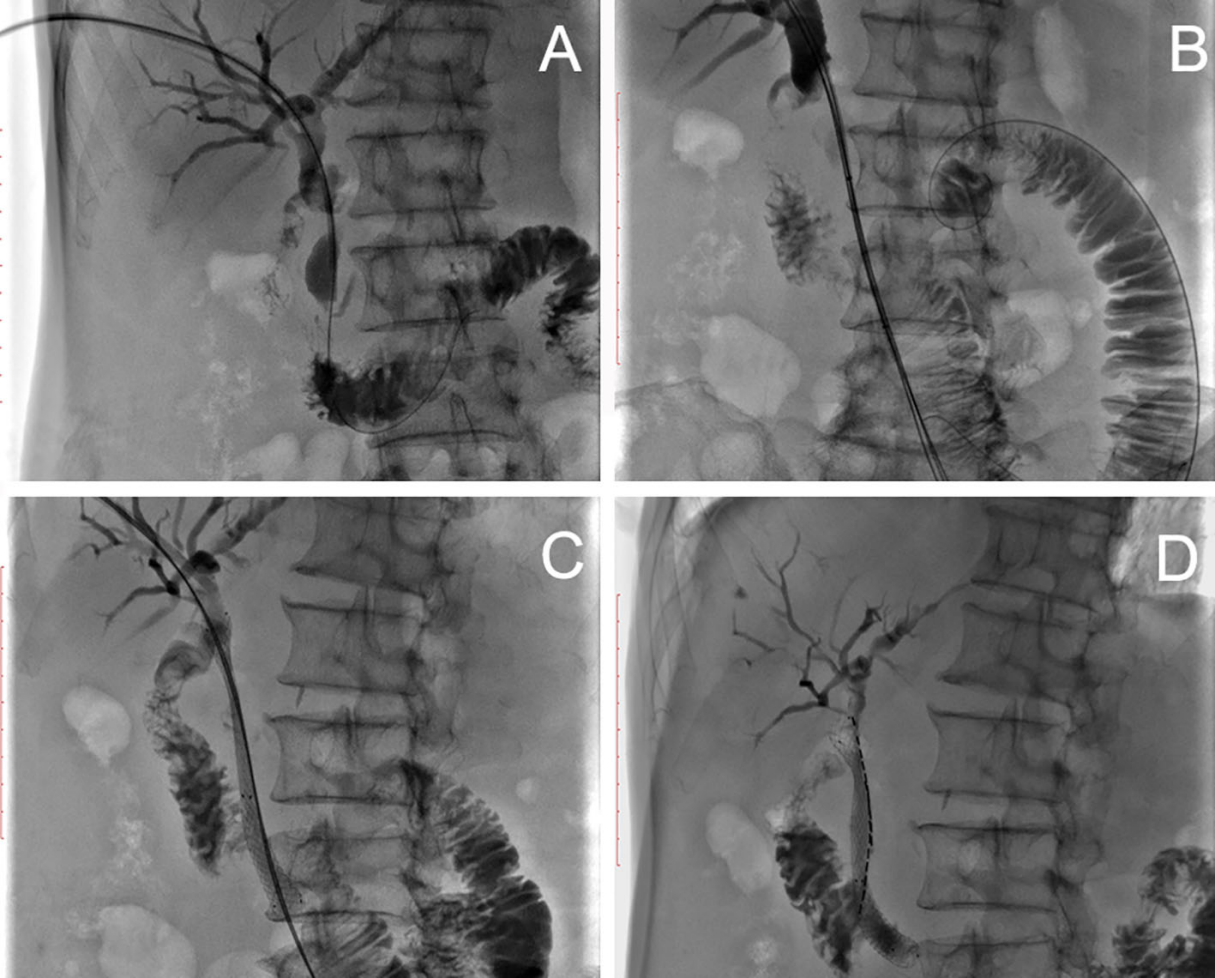
Patient with MOJ. **(A)** Cholangiogram showing dilatation of the intrahepatic bile ducts and the initial common bile duct and severe stenosis of the common bile duct. **(B)** Two 0.018-inch hydrophilic guidewires and a stiff wire were carefully advanced through the stenotic segment into the distal duodenum. **(C)** After stent placement, two 0.018-inch wires were left outside the stent to introduce a seed strand. **(D)** Two seed strands were implanted over two 0.018-inch wires each.

**Figure 2:**
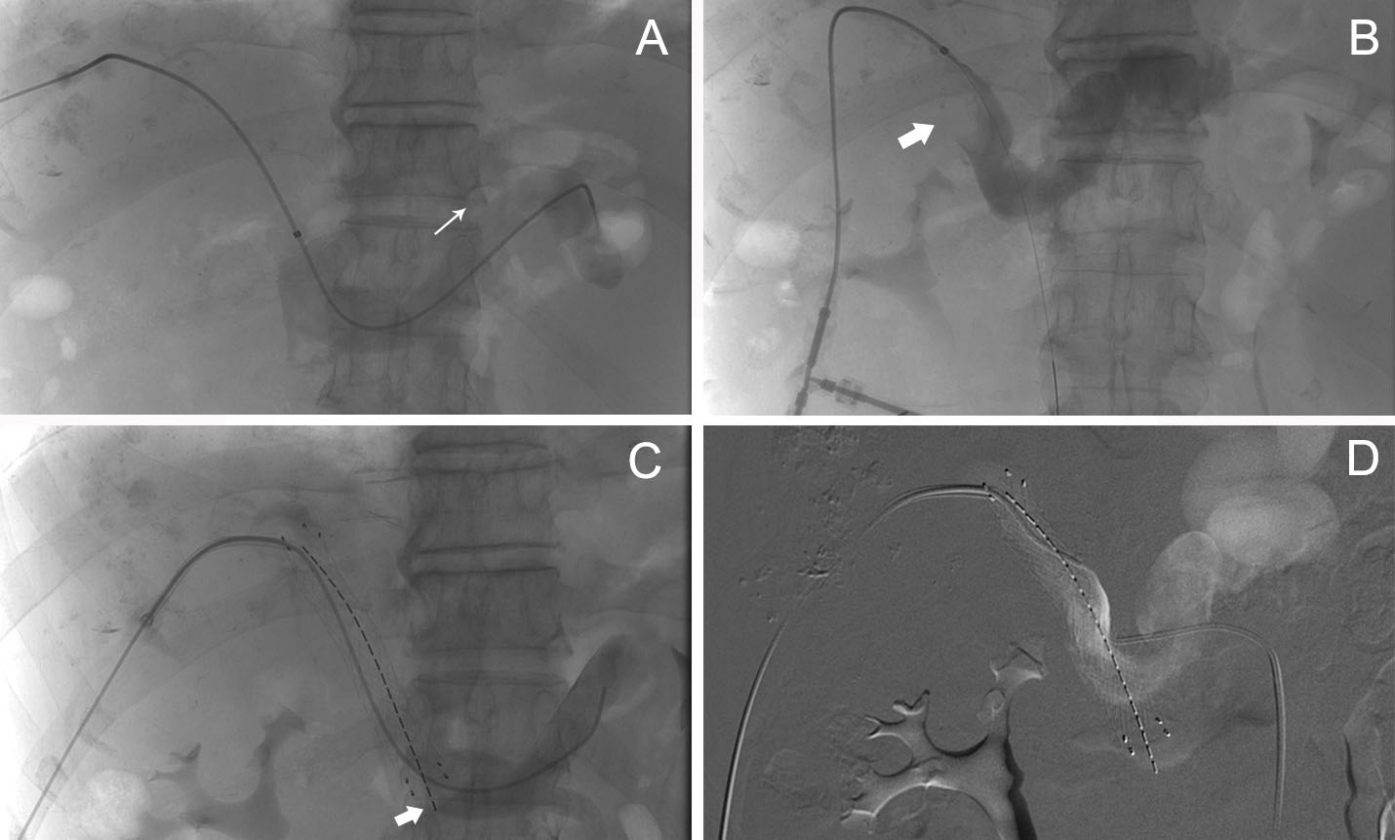
A patient with PVTT. **(A)** Angiography of the splenic vein showed blood flow away from the liver, the direction corresponding to the arrow. **(B)** The obstruction was located at the main portal boot (arrow). **(C)** After implantation of a self-expandable stent and an iodine-125 seed string under DSA, the seeds moved in the tube as the patient’s position changed, causing internal irradiation of normal tissue (arrow). **(D)** Finally, portal venography showed patency of the stent.

In group A, implantation of iodine-125 seed strands in combination with stent placement was successfully performed in all patients, and there was no technical failure during implantation of the seed strands and placement of the stent (**Figures 1**, **2**). The technical success rate of this procedure was 100%. A self-expandable nitinol stent was implanted in each patient. In group A, an average of 1.07 ± 0.26 strands (range of 1 to 2 strands) with an average of 11.89 ± 4.1 (range of 8 to 22 seeds) iodine-125 seeds were implanted in each patient. In all patients there was no displacement of the stent. Only in one patient with PVTT a small displacement of the seed strand was observed (**Figure 2**). In group B, stent placement was successful in all patients. Patients received TACE an average of 3.4 times in group A and an average of 3.11 times in group B (P=0.311).

There were no complications during or after treatment and no deaths in connection with the operation.

**Figure 3:**
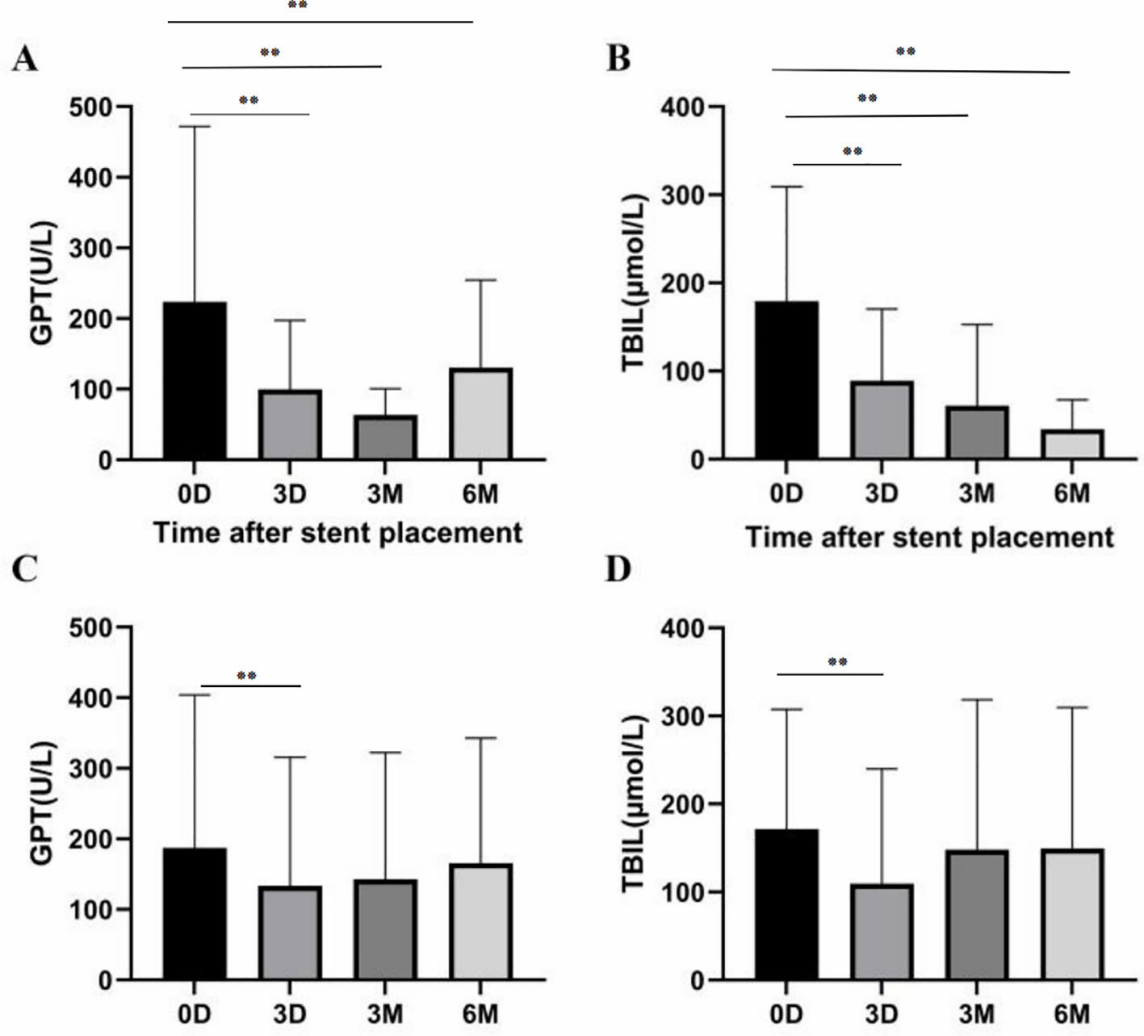
**(A, B)** In group A, there were significant differences in GPT and TBIL between baseline, 3 days, 3 months and 6 months later (P<0.01, respectively). **(C, D)** In group B, GPT and TBIL levels 3 days later were lower than at baseline (P<0.01), while GPT and TBIL levels 6 months later were similar to baseline (P=0.07 and P=0.45, respectively). *P<0.05, **P<0.01.

Compared to pre-surgery, total bilirubin (TBIL) and glutamate pyruvic transaminase (GPT) were Significantly improved 3 days after surgery (**Figure 3**).

### 3.3 Follow-up

During follow-up, TBIL and GPT in group A patients showed a continuous improvement over six months compared to pre-treatment results (P<0.001). However, the reduction in TBIL and GPT levels could not be maintained in group B six months after treatment when compared to pre-treatment results (P=0.07) (**Figure 3**).

**Figure 4:**
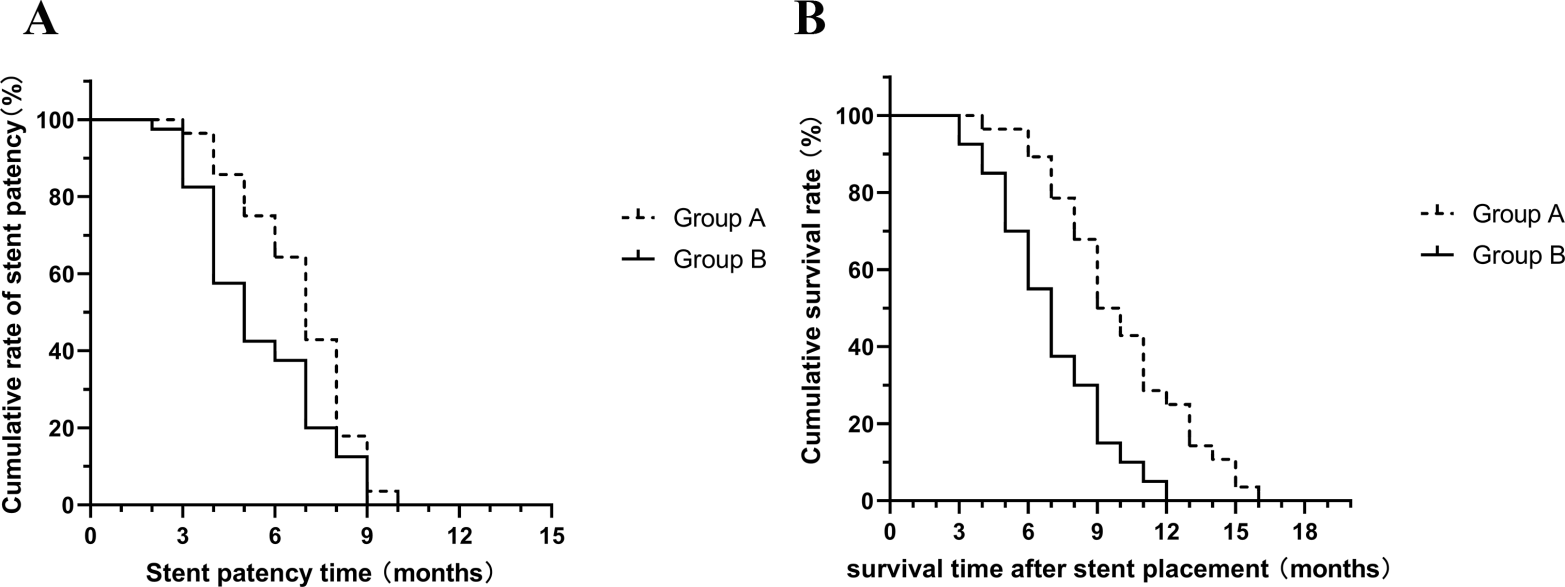
**(A)** Mean stent patency time was significantly longer in group A than in group B (P=0.032); **(B)** Mean survival time was significantly longer in group A than in group B (P<0.001).

The mean stent patency in group A was 6.86 ± 1.82 months and the mean survival time was 10.03 ± 3.04 months. In group B, the mean stent patency was 5.5 ± 2.09 months and the mean survival time was 7 ± 2.44 months (**Figure 4**). Both results showed that patients in group A were able to achieve longer stent patency and survival time (P=0.032, P<0.001).

## 4 Discussion

Malignant lesions located in the porta hepatis were not suitable for surgery due to invasion and metastasis. Stent implantation was a safe palliative treatment aimed at removing the obstruction, improving the general condition of patients and reducing suffering (18, 19). But this surgery had a high rate of stent restenosis due to the invasive growth of the tumor (20–22). To control the local invasion of the tumor, various methods such as TACE, combined sorafenib chemotherapy, external beam radiotherapy and internal beam radiotherapy have been tried.

However, in patients with poor general health (especially poor liver function), some results have shown that simple TACE could not control tumor invasion and also caused liver damage (23, 24). In hypovascular tumors, TACE could not achieve an ideal effect due to the poor blood supply to the tumors. Intensity-modulated radiotherapy could be an option for PVTT or MOJ, and again TACE was required to control tumor invasion into the liver or other organs (25, 26). For peripheral organs surrounding tumors that are sensitive to radiotherapy, radiotherapy could cause damage. It can cause damage to the liver parenchyma and minor damage to the bile duct intima (27). In chemotherapy, high TBIL and poor liver function limited this treatment. Sorafenib is an oral multi-kinase inhibitor. Two large, randomized, controlled, international, multicenter phase III clinical trials have shown that sorafenib can delay tumor progression and prolong survival in patients with advanced HCC (33). Sorafenib was a kind of targeted agent for HCC and had a good effect together with TACE (28). But sorafenib was too expensive, and it was not suitable for all patients.

Iodine-125 seed was a type of low-level radioactive source. It was ideal for internal radiotherapy in clinical practice (29). In order to control tumor invasion into the biliary tract, G.J. Teng developed a stent loaded with iodine-125 seed for biliary tract irradiation. The study used the method of internal and external stent nesting, that is, the outer scaffold containing radioactive I125 particles was placed in the target area, and then the inner scaffold was inserted into the expanded outer stent nest. The therapeutic effect of internal radiation was thus achieved. It did not aggravate the patient’s suffering (30). With this type of stent, a satisfactorily efficient drainage of bile was achieved (31). This type of stent has not yet been widely used in patients with PVTT, and in some countries this stent has not yet been fully commercialized. The implantation of iodine-125 seed strands in combination with stenting and TACE was invented by Z.P. Yan, and this type of new therapy has achieved a satisfactory clinical effect. Their results show that this new therapy can prolong the survival time of patients and significantly reduce the incidence of disease progression (12, 14). In agreement with the previous studies, the results of our study also suggest that ILBT in combination with stent implantation and TACE can prolong stent patency and survival time, in contrast to simple stent implantation and TACE. In view of the satisfactory effect, this therapy was worth popularizing. To perform the operation successfully, high surgical skills were required, especially for the preparation and implantation of the iodine-125 seed strands.

A 4F catheter was perfect to contain seeds linearly. The Hyalin catheter was the better option as the number and position of the seeds could be easily confirmed visually (32). In practice, the introducer of the 6F NEFF set was the more convenient choice, and the number and location of seeds could be confirmed under DSA. After all seeds were loaded, the stiffening cannula of the 6F NEFF should be carefully withdrawn in case seeds were accidentally pulled out. When sealing the remaining end, the redundant part of the catheter should be left as short as possible in case the seeds move in the catheter when the patient’s position changes. Moving the seeds would result in the internal irradiation occurring in the wrong place. In our experience, half the seed length was perfect for sealing.

Due to stent restenosis usually occurring at the ends of the stent, both ends of strand and stent should adequately extend beyond the edge of the lesion, and the length should be shorter than 10 mm in case the iodine-125 seeds cause radioactive damage to normal tissue. The irradiation radius of one strand was usually sufficient to completely cover the lesion. In some patients, however, one strand was not able to absorb all iodine-125 seeds. In these cases, multiple strands were needed to load all iodine-125 seeds. These strands should be placed next to each other around the center of the lesion. Prior to stent placement, two 0.018-inch guidewires were required to advance through the obstruction, so the inner diameter of the sheath should be large enough. the 5F sheath already met the requirements, and only if necessary could a larger size of sheath be used. A larger size of sheath could cause injury to the lumen or hemorrhage when advancing. For instance, S. L. Yang et al. reported 17 cases of primary liver cancer complicated with portal vein thrombosis were treated with the iodine-125 seed strands in combination with stent implantation. They used a 6F catheter sheath (Cook company) and a 5F guide to insert the iodine-125 seed strands during surgery, and the patients had no obvious shortness of breath, pain and bleeding during and after surgery. Similarly, in the study by LI C X et al., a 6F catheter sheath and 0.035 double stiffened guidewires were used for the insertion of the iodine-125 seed strands and portal stents, and satisfactory results were obtained in terms of technical safety and feasibility. The puncture tract is closed with an embolic coil (34, 35).

The liver had the ability to heal itself. After implantation, an absorbable gelatin sponge was used to close the transhepatic puncture track. The gelatin sponge would be absorbed within 2–3 weeks, and in this time most of the puncture track healed. In our opinion, the resorbable gelatin sponge was a better choice than coil. This was different from previous reports (11). During the follow-up after particle implantation, no complications related to radiation damage such as hemorrhage, infection, pain or damage to surrounding organs were observed in any of the patients in the treatment group. In previous relevant reports, only a few studies have observed severe radiation damage from iodine-125 seeds in the treatment of liver cancer with lymph node metastases, which also confirms the high safety of iodine-125 seed implantation surgery in the treatment of abdominal solid tumors (36, 37).

Our study has some limitations. The number of samples in the group was relatively small due to the limited number of cases in which poor general health status, including Child-Pugh class C, ECOG score ≥ 3, life expectancy<3 months, Manager’s Difficulty, can be excluded. In addition, the patient samples are from a single center, which makes our study less representative. Due to these limitations, we cannot draw far-reaching conclusions from the results. For this reason, some of our findings need to be confirmed in multicenter studies with a larger sample size.

This study is a 6-month short-term efficacy follow-up, with relatively limited observational data focusing mainly on liver function and stent patency. There was no long-term follow-up of the tumor itself and the patients’ quality of life. The half-life of the iodine-125 seeds is approximately 60 days. After three half-lives, there are no studies on its own radioactivity and effect in tumor treatment or whether it is necessary to supplement the implantation of iodine-125 seeds.

Currently, the cost of stents and iodine-125 seeds is relatively expensive and most people do not opt for this comprehensive treatment. Instead, they opt for a relatively inexpensive treatment or a single treatment, such as percutaneous transhepatic biliary drainage or iodine-125 seed strands only. Taken together, the implantation of iodine-125 seed strands in combination with stent implantation and TACE is a safe and effective treatment for MOJ and PVTT. This therapy was able to continuously improve jaundice and liver function over a relatively long period of time, and the improvement in jaundice and liver function helped to significantly reduce the incidence of disease progression. Although this is a retrospective control study with limited evidence, we believe that our results provide a good indication for designing further studies.
